# Cilium-autonomous regulation of tubulin transport by IFT

**DOI:** 10.1186/2046-2530-4-S1-O8

**Published:** 2015-07-13

**Authors:** K Lechtreck

**Affiliations:** 1 Cellular Biology, University of Georgia, Athens, GA, USA

## Objective

The assembly of the axoneme, the structural scaffold of cilia and flagella, requires translocation of a vast quantity of tubulin into the growing cilium, but the mechanisms that regulate the targeting, quantity, and timing of tubulin transport are largely unknown.

## Methods

GFP-tagged α-tubulin was expressed in *Chlamydomonas reinhardtii *and its transport in cilia was analyzed using TIRF microscopy.

## Results

GFP-tagged α-tubulin entered *Chlamydomonas *cilia as a cargo of IFT and by diffusion. IFT-based transport of GFP-tubulin occurred at a low frequency in full-length steady-state cilia and was strongly increased during ciliary growth when IFT trains carried more tubulin. Cells possessing both non-growing and growing cilia selectively targeted GFP-tubulin into the latter indicating that cells regulate tubulin influx individually for each cilium. The preferential delivery of tubulin boosted the concentration of soluble tubulin in the matrix of growing cilia. Cilia length mutants showed abnormal kinetics of tubulin transport, suggesting that ciliary length control involves a regulation of the occupancy of IFT trains by tubulin cargoes.

## Conclusions

Tubulin is a *bona fide *cargo of IFT. We propose that IFT functions as a tubulin pump concentrating soluble tubulin in growing cilia which promotes the elongation of the axonemal microtubules and ciliary growth.

